# Local buckling of cold-formed steel trapezoidal sheets: Data for finite element model validation

**DOI:** 10.1016/j.dib.2024.110075

**Published:** 2024-01-18

**Authors:** Miquel Casafont, Frederic Marimon, Oriol Bové, Miquel Ferrer, Xavier Centelles

**Affiliations:** Research Group in Structures and Mechanics of Materials (REMM), Department of Strength of Materials and Structural Engineering, Universitat Politècnica de Catalunya (BarcelonaTech-UPC), Avinguda Diagonal 647 09028, Spain

**Keywords:** Cold-formed steel structures, Cold-formed steel sheeting, Finite element modelling

## Abstract

Data is provided from a validation example for a finite element model of a cold-formed steel trapezoidal sheet. The sheet is subjected to bending, failing due to local buckling. The numerical model and the validation procedure are carried out according to the new Eurocode 3 prEN1993-1-14 Design assisted by finite element analysis. Detailed information concerning all aspects needed to reproduce the example is included: (i) the nominal and measured values of the sheet geometry; (ii) the measured material properties of the steel; (iii) the test setup of the validation experiments; (iv) the experimental results; (v) a complete description of the finite element model and solution procedure; and (vi) the finite element results. Additionally, data related to sensitivity studies on the numerical model is also presented, including the effect of the model domain, meshing, and imperfections (shape, magnitude, direction and combinations). Overall, the article aims to provide data and guidance to designers and researchers validating similar numerical models.

Specifications Table

**Table:** 

Subject	Engineering
Specific subject area	Civil and Structural Engineering
Data format	Analyzed
Type of data	Table, Image, Graph, Figure
Data collection	The experimental data was obtained from bending tests conducted following the Annex A of Eurocode 3 EN 1993-1-3 [1]. The numerical model, and the verification and validation procedures were carried out according to the Eurocode 3 prEN1993-1-14 [2]. The model was developed using ANSYS mechanical APDL Release 19.0 [3] on a computer with an Intel Core i5-10600 CPU 3.30 GHz.
Data source location	The experimental campaign was conducted in the Laboratory of Mechanical Tests of AIDIMME Instituto Tecnológico, Paterna, Spain. The numerical simulations were performed in the REMM research group of the Department of Strength of Materials and Structural Engineering at the Universitat Politècnica de Catalunya (UPC-BarcelonaTech), Barcelona, Spain.
Data accessibility	Repository name: CORA. Repositori de Dades de Recerca Data identification number: 10.34810/data899 Direct URL to data: https://dataverse.csuc.cat/dataset.xhtml?persistentId=doi:10.34810/data899

## 1 Value of the Data

• The experimental data from the validation test can be used in other analogous finite element model validation procedures.
• The numerical model provided, along with its description, can serve as a guide for finite element modelling of sheets in similar situations. Noticeably, while there have been numerous investigations applying finite element modelling to cold-formed steel structures, including trapezoidal sheeting in general, specific studies focusing on sheets subjected to pure bending are scarce, see [4].
• The provided data aims to illustrate the application of the forthcoming EN1993-1-14 [2] to cold-formed steel structures, with a particular focus on numerical model development for trapezoidal sheeting, and verification and validation procedures.
• Researchers, designers and manufacturers of cold-formed steel sheeting can benefit from the data presented herein. In Europe, the bending strength of the sheets has traditionally been determined following the standard EN1993-1-3 [1,5], which involves using elaborated hand calculation approaches and costly experiments. The proposal of a standardized procedure for a finite element-based design approach can help in several aspects, such as reducing the number of experimental tests, improving predictions of the hand calculations and developing better calculation procedures.

## 2 Objective

The primary objective of these data is to illustrate the development, verification and validation processes involved in creating a finite element model for a cold-formed steel sheet, following the standard EN1993-1-14 [2,6]. Furthermore, the experimental and numerical data provided can directly be used to verify and validate other numerical models intended for similar applications.

It is worth mentioning that there are similar experimental data compiled in [7,8], which can complement the test results included here, as well as other numerical models for determining the bending strength of cold-formed steel sheets in [4,^6^,^8^,9].

## 3 Data Description

This section contains the basic data needed to reproduce the validation example:

1. Nominal geometry of the cross-section (Fig. 1). The nominal mid-surface geometry of the sheet shown in Fig. 1 is used to create the shell finite element model, as recommended in [6]. It should be pointed out that two types of dimensions are defined in the Figure: (i) distance between intersection points of section elements, and (ii) corner radii. During the analysis process, this nominal geometry is modified according to prescribed imperfections (see Section 4.5 for their complete description).

**Fig. 1:**
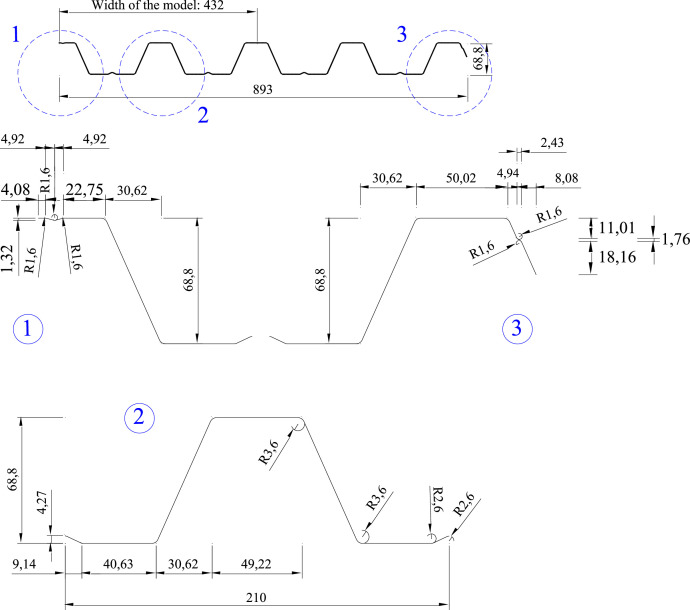
Cross-section nominal dimensions (in mm).
2. Measured sheet thickness: 0.695 mm. The experimentally measured core sheet thickness is applied to the shell model. This value is obtained from the coupons utilized in the material tests and corresponds to the mean value measured in 4 different coupons.
3. Measured material stress-strain curve (Fig. 2, Table 1). The material curve of the numerical model is defined from the average of the experimental material curves obtained from the coupon tests. Fig. 2 includes the experimental (Fig. 2a), average (Fig. 2a, b) and FEM (Fig. 2b) material curves. Table 1 contains the stress-strain couples of the average material curve used in the finite element model.

**Fig. 2:**
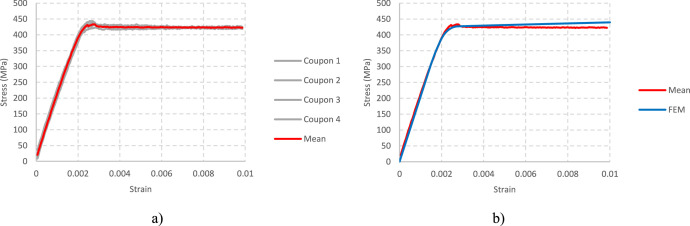
Material curves: a) experimental; b) experimental mean curve vs. numerical curve.

**Table 1:** Stress-strain couples of the FEM material curve.

	1	2	3	4	5	6
Strain	0	0.00145	0.00150	0.00170	0.00190	0.00197
Stress (MPa)	0	298	308	344	375	385
	7	8	9	10	11	
Strain	0.00213	0.00232	0.00254	0.00277	0.01000	
Stress (MPa)	402	415	424	427	440
4. Experimental setup (Fig. 3, Fig. 4). The validation experimental tests are conducted according to the EN1993-Part 1.3 [1]. Fig. 4 shows the experimental test setup, including the longitudinal dimension of the tested specimens, location of supports and forces, support conditions, force application system, measuring devices and auxiliary elements (wood blocks and transverse ties). The numerical model is created using the nominal dimensions shown in Fig. 4.

**Fig. 3:**
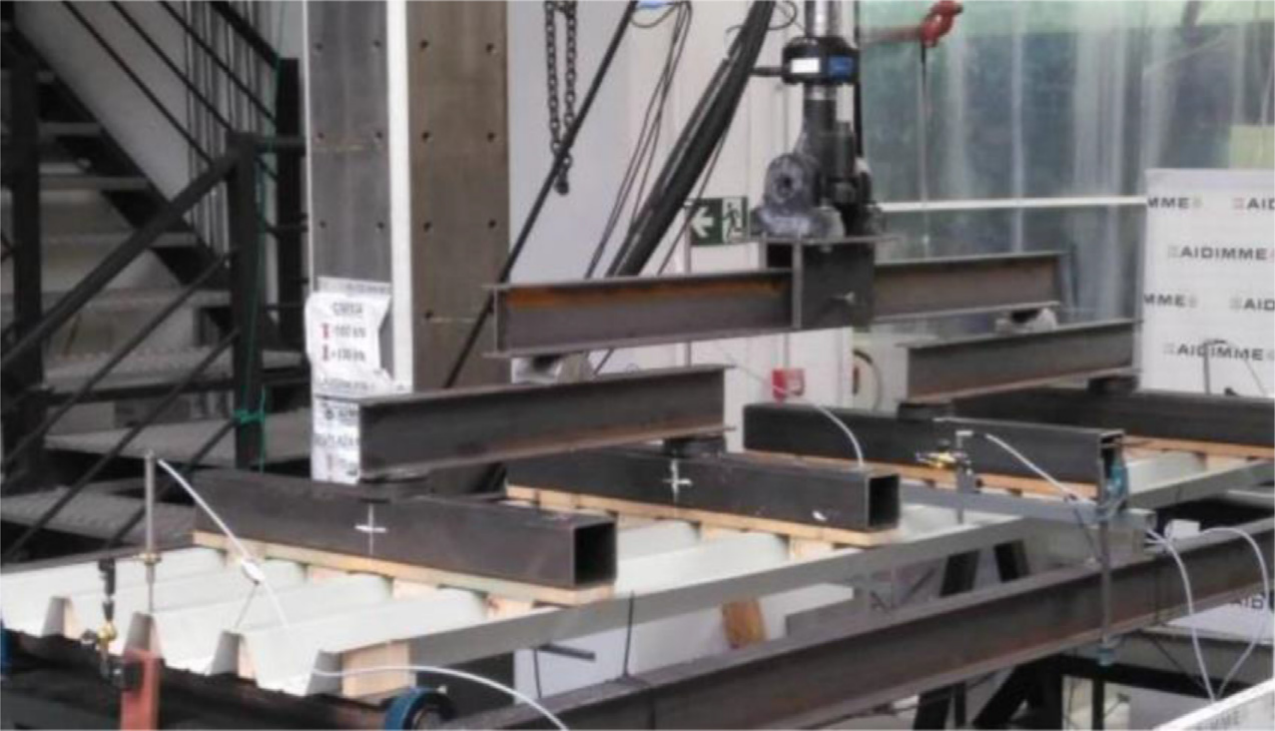
Specimen being tested.

**Fig. 4:**
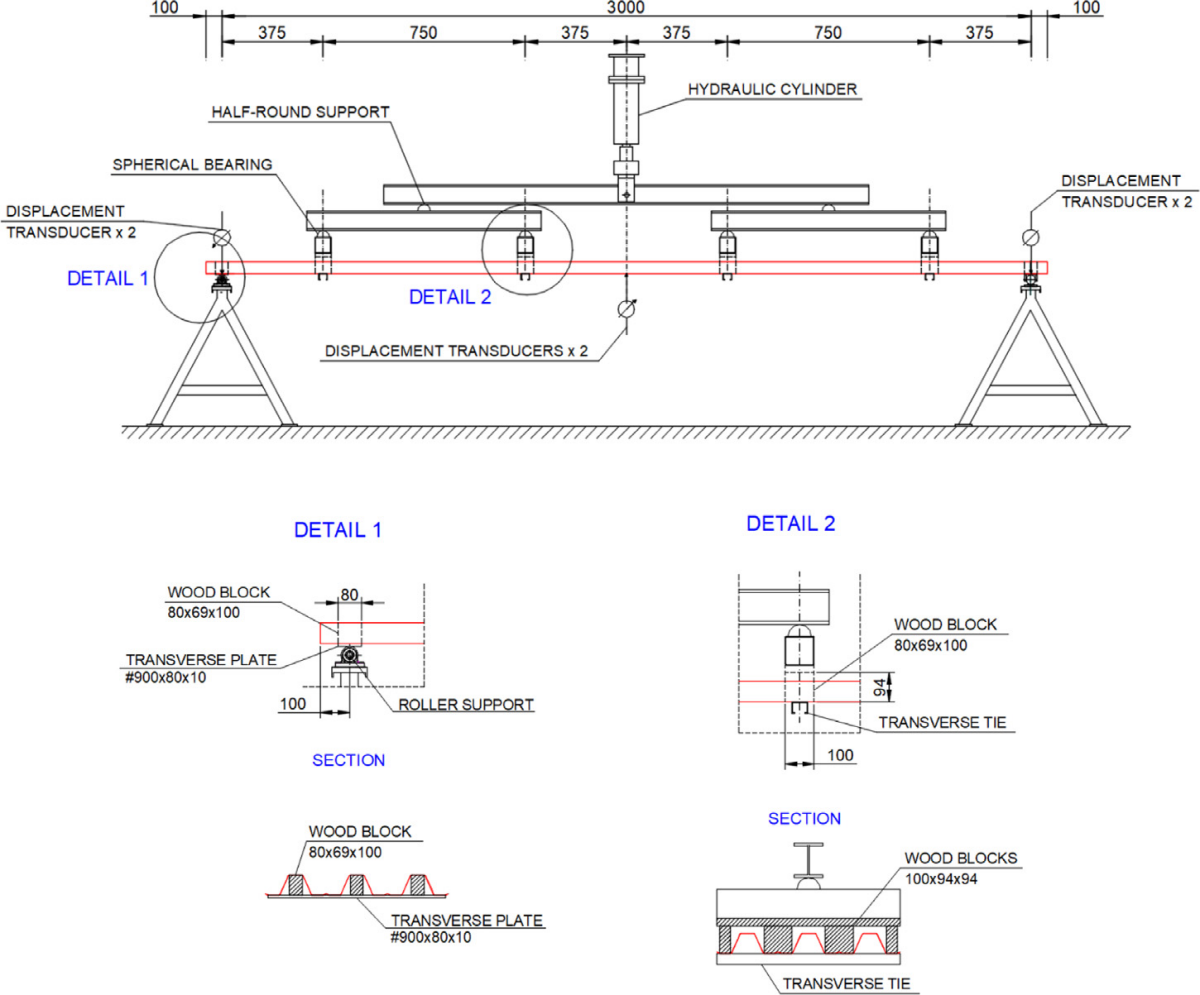
Test setup (dimensions in mm).
5. Numerical model (Fig. 5). A complete discussion on the derivation, characteristics and solution of the numerical model is included in Section 4 of the article. The model finally utilized in the validation example is displayed in Fig. 5.

**Fig. 5:**
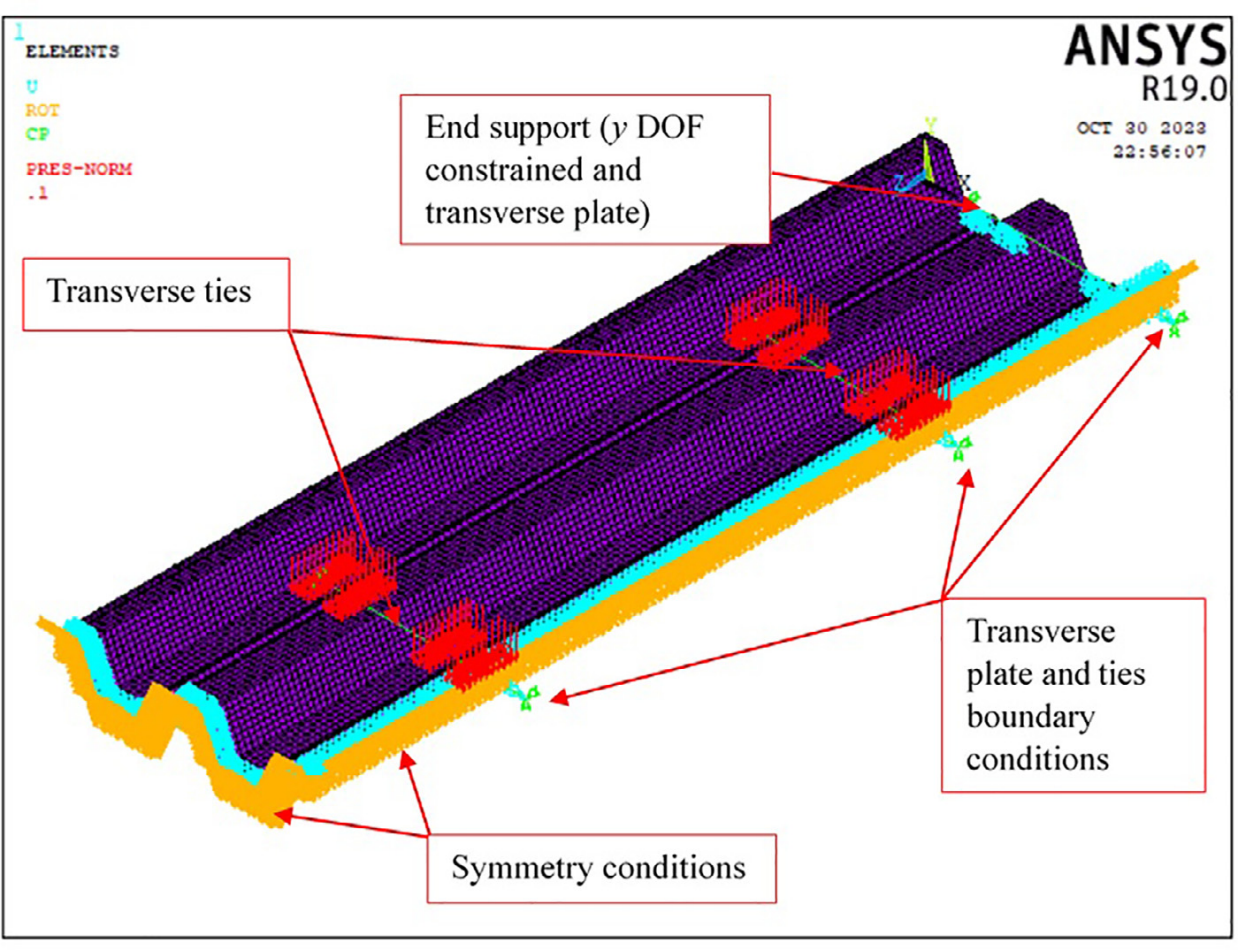
Finite element model with boundary conditions and applied pressure.
6. Experimental and numerical values of ultimate force (Table 2). The ultimate force is the system response quantity (SRQ) chosen for validation. The experimental and numerical ultimate forces are included in Table 2, together with other relevant results.

**Table 2:** Experimental and numerical ultimate force.

Experimental		Numerical		Ratios		
Specimen	F_test_ (N)	Imperfection combination (see Section 4.5)	F_FE_ (N)	F_test_/ F_FE 1-2-3_	F_test_/ F_FE 2-3-4_	F_test_/ F_FE 3-4-5_
1	9559.0	1-2-3	9850.69	0.97	0.97	0.96
2	9873.9	2-3-4	9825.76	1.00	1.00	0.99
3	9983.0	3-4-5	9970.57	1.01	1.02	1.00
Average	9805.3			1.00	1.00	0.98
7. Experimental and numerical force-displacement curves (Fig. 6). Complementary to the SRQ, the force-displacement curves shown in Fig. 6 are also useful for validation purposes.

**Fig. 6:**
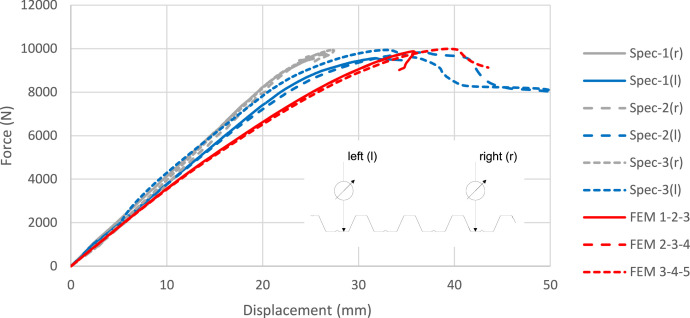
Experimental and numerical force-displacement curves.
8. Experimental and numerical failure modes (Fig. 7). Finally, the agreement between the experimental and the numerical failure modes can be observed in Fig. 7.

**Fig. 7:**
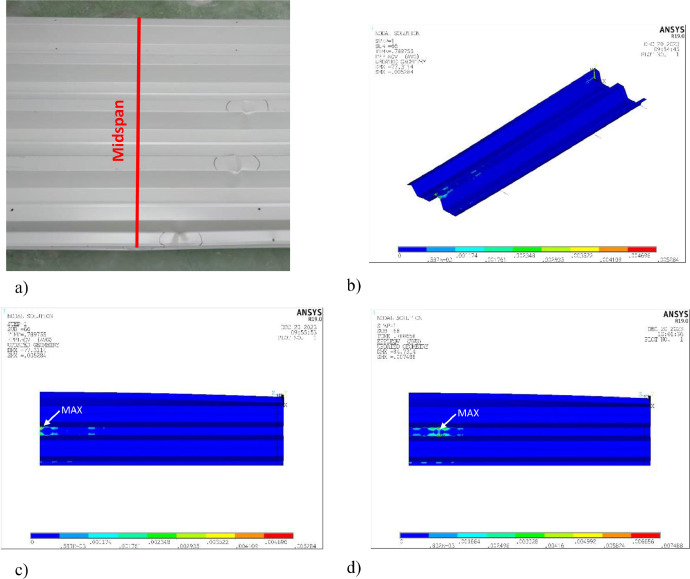
Experimental and numerical failure mode: a) experimental local buckling; b) and c) numerical failure when using 1-2-3 imperfection combination (von Mises plastic strain); and d) numerical failure with 3-4-5 imperfection combination (von Mises plastic strain).

## 4 Experimental Design, Materials and Methods

### 4.1 Model domain and geometry

The model includes one quarter of the tested specimen, as depicted in Fig. 5. Although the transverse cross-section of the sheet is slightly asymmetrical (Fig. 1), it was observed in the verification stage that modelling the left half produces almost the same results as modelling the whole section (see Section 4.7.1). On the other hand, the test setup is symmetrical in the longitudinal direction; consequently, it makes sense to consider only half the specimen length.

It should be pointed out that the model is created according to the geometry of Fig. 1, including the rounded corners as recommended in [2,6].

The model domain is limited to the specimen itself; that is, neither the force application system nor the supports are realistically represented. Contrarily, the transverse support plate and ties (Fig. 4), which prevent the spreading of the longitudinal corrugations [1,5], are included.

### 4.2 Model discretization

The trapezoidal sheet is discretized using quadratic shell finite elements (Ansys shell 281). The mesh density is defined on the basis of the mesh sensitivity study presented in the verification Section (4.7.2). The finite element mesh is uniform in all the model with a size of 10 mm. This element size is a reference value used by the mesh generator. The actual size may vary slightly to ensure the element conforms to the varying geometries within the model's components. The thickness of the shell element is equal to the measured thickness of the sheet.

The mesh sensitivity study also confirms that one quadratic shell finite element is enough in the corners of the cross-section.

The transverse support plate and ties are modelled with a single spar finite element (Ansys link 180). Their nominal cross-sections are 800 and 48 mm^2^, respectively. It is noted that the simplified model of the support plate only aims to reproduce its effect on the transverse deformation of the sheet. It is not intended to accurately model the support behavior (see next Section).

### 4.3 Support and load conditions

The region of interest of the tested specimens is the central part (the lower left part of the model shown in Fig. 5), where failure occurs. This is taken into account when modelling the supports, that are located far from this region. Their modeling is simple, consisting of a line of constraints that restrain the *y* degree of freedom (DOF) of the nodes located at the lower flange of the end section (see Fig. 5). On the other hand, the model also includes the constraints needed to simulate the longitudinal and transverse symmetry conditions.

The transverse plate and ties constrain conditions should also be indicated: at one end, the spar element is directly connected to one of the shell nodes; while at the other end, the *x* DOF is constrained, to simulate the symmetry, and the *y* and *z* DOFs are coupled to the corresponding DOFs of the opposite end (i.e. to the DOFs of the shell node where the spar element is connected).

The load is directly applied through a distributed pressure on the lower flange of the sheet (Fig. 5). The location and area of the pressure application surface correspond to the nominal contact surface between the wood blocks and the sheet (Fig. 4). The same pressure force is applied in all the analyses: *p*=0.1 MPa. The ultimate strength falls within 6/10 and 8/10 of this pressure value.

### 4.4 Material models

The material model is defined by the multilinear stress-strain curve specified in Table 1, with isotropic hardening and Von Mises Yield criterion. For strains higher than the last value given in Table 1, the corresponding stress is determined according to the slope of the last curve segment. This slope is set to 1°, slightly higher than the experimental one, to ensure numerical stability as proposed in [2].

The first part of the material curve is considered linear until 70% of the measured yield stress, the second couple in Table 1 (the measured 0.2% yield stress is 425 MPa). The experimental elastic modulus is defined from this couple: 205517 MPa. The Poisson ratio is taken equal to 0.3 [5].

It should be noted that a 40 mm segment from the end support of the sheet in the longitudinal direction is modeled with linear material behavior. This is done to prevent localized yielding of the sheet ends caused by the simplified modelling of the supports. As discussed above, the end supports are modelled constraining vertical displacements along a single line at the lower flange of the sheet. As a consequence, a significant stress concentration arises (since there is a numerical singularity), causing premature and non-realistic yielding. Once the concentrated yielding initiates, the non-linear analysis fails to progress beyond this point due to convergence issues. This occurs in the model well before reaching the expected ultimate bending strength. Although accurate modeling of the end support conditions could resolve the problem, it would unnecessarily complicate the analysis. This would necessitate the inclusion of a more sophisticated model of the end plate and the corresponding contact elements with the sheet. Instead, the preferable approach is to use the simple linear support and turn the material behavior to linear

Material linear behavior is also applied to the transverse plate and ties.

### 4.5 Imperfections

Only geometric imperfections are considered in the validation process. Neither the residual stresses nor the enhancement of the yield stress due to cold work are included in the model. This approach aligns with the guidelines set by [6] for numerical simulation and validation of the cold-formed steel members covered by Eurocode 3 Part 1- 3 [5].

**Fig. 8:**
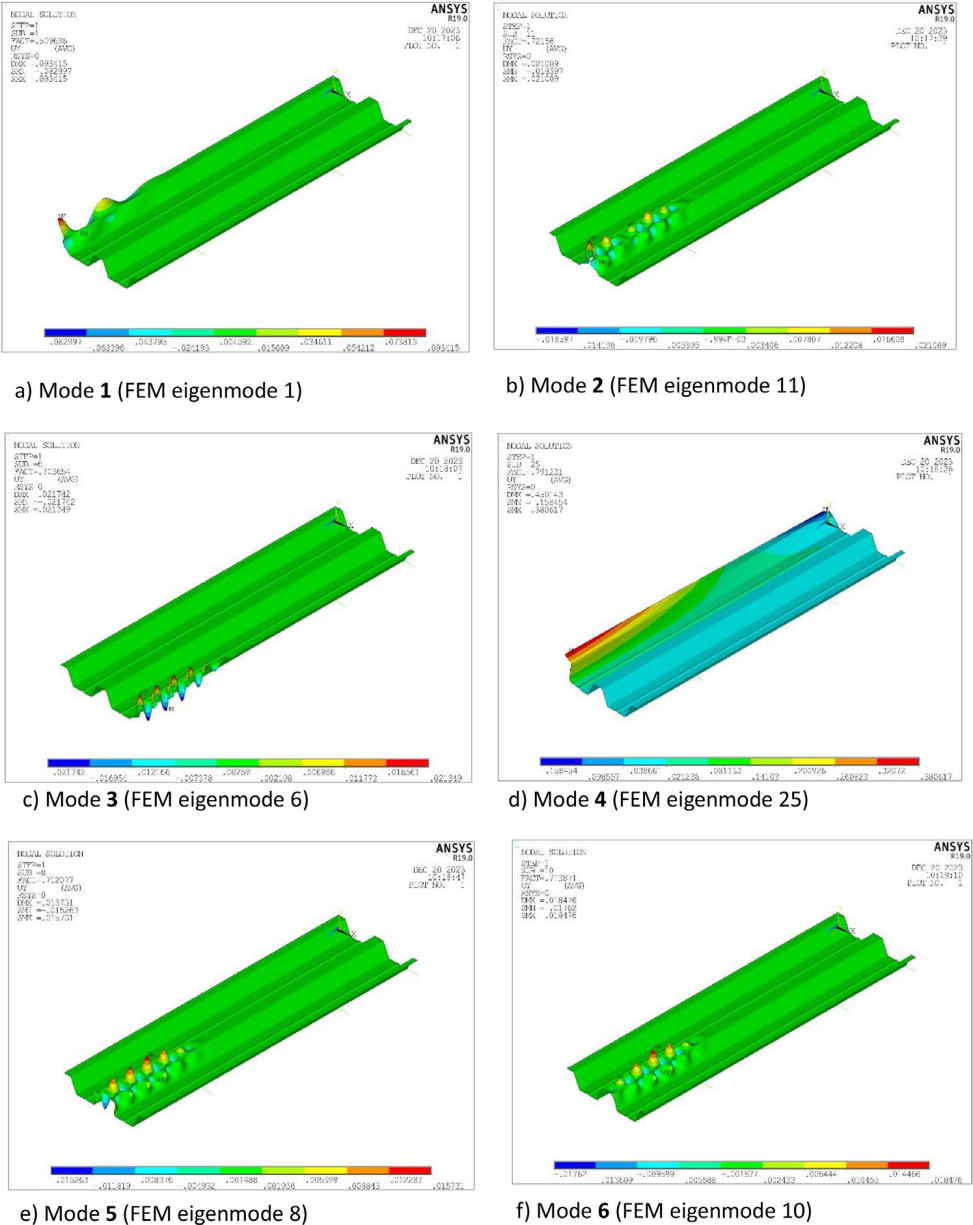
Imperfection modes considered in the study.

The imperfection shapes correspond to eigenmodes determined in a linear buckling analysis (LBA). Fig. 8 displays the imperfection shapes used in the present model. The first three are local modes (from now on called modes 1, 2 and 3), while the fourth is a distortional mode (called mode 4). A distortional imperfection is considered because the failure mode observed in the initial simulations showed a small participation of such buckling mode deformation. Concerning the local modes, it is noted that they are the lowest ones provided by the LBA showing local buckling in the central cross-section of the sheet. Fig. 8 also includes the eigenmode number resulting from the buckling analysis. It is noticeable that the local buckling modes do not occur simultaneously in the three modelled longitudinal ribs.

An imperfection sensitivity study is conducted in Section 4.7.3, which includes analyses with: (i) the individual imperfections shapes shown in Fig. 8, considering two directions (upward and downward buckle), (ii) combinations of these 4 imperfections, and (iii) combinations with other relevant local mode imperfections (modes 5 and 6).

Imperfections were not measured in the specimens before testing. In this situation, [6] recommends the use of the following code proposed imperfection magnitudes [2]:

1. Local imperfection mode for ribs 2 and 3 (modes 2 and 3 in Fig. 8): *e_0,loc_*=*b*/200, where *b* is the notional flat width of the upper flange element.
2. Local imperfection mode for rib 1 (mode 1 in Fig. 8): *e_0,loc-out_*=*b*/125. This value was proposed in [2] for outstand elements of members, but it is tested here for sheeting. It is also noted that due to the presence of the longitudinal stiffener in the upper flange of the first rib (Fig. 1), it could be considered that buckling mode 1 is distortional and, consequently, a distortional imperfection magnitude should be applied. However, given the small dimensions of such a stiffener and its limited impact on the buckling of rib 1 (see Fig. 8), it is reasonable to treat it as a local mode.
3. Distortional imperfection mode for rib 1 (mode 4 in Fig. 8): $e_{0,dist}=0.3\cdot t\cdot \sqrt{M_{y}/M_{cr,d}}$, where t is the core thickness of the sheet, *M*_y_ is the yield moment of the cross-section, and *M*_cr,d_ is the distortional buckling moment. The latter was determined from the signature curve derived with the finite strip method using the CUFSM program [10].

Tables 3 and 4 show the resulting imperfection magnitudes, together with some data needed for their derivation.

**Table 3:** Local imperfection magnitudes.

Mode	Shape (Fig. 8)	Equation	b (mm)	*e*_0_ (mm)

Local rib 1	1	b/125	32.23	0.2578
Local ribs 2 and 3	2.3	b/200	48.46	0.2423

**Table 4:** Distortional imperfection magnitude.

Phase	Mode	Shape (Fig. 8)	t (mm)	fy (MPa)	My (Nmm)	Mcr (Nmm)	*e*_0_ (mm)

Verification (from nominal values)	Distortional rib 1	4	0.71	280	2257644	999449	0.32
Validation (from measured values)	Distortional rib 1	4	0.695	425	3426700	977395	0.39

### 4.6 Solution scheme

The analysis includes geometric and material nonlinearities. It is solved using the arc-length technique, considering initial step sizes in the range of *p*/120 and *p*/30 (where *p* is defined in Section 4.3), and maximum and minimum radius multipliers of 1 and 0.0001.

### 4.7 Verification

Unless stated otherwise, the verification analyses are carried out on a model that considers a quarter of the specimen, a mesh with an element size of 10 mm, and an imperfection resulting from the superposition of modes 1, 2, and 3 (Fig. 8). Verification was conducted before validation (i.e. before working on the experimental results), as recommended by [11]. Consequently, the following nominal values were applied to the model: nominal core sheet thickness, 0.71 mm; and nominal material properties: bi-linear stress-strain curve with *f*_y_=280 MPa, which corresponds to the sheet steel grade S 280 GD, elastic modulus *E*=210000 MPa, and tangent elastic modulus *E*_t_=2100 MPa [2].

#### 4.7.1 Model domain selection

**Fig. 9:**
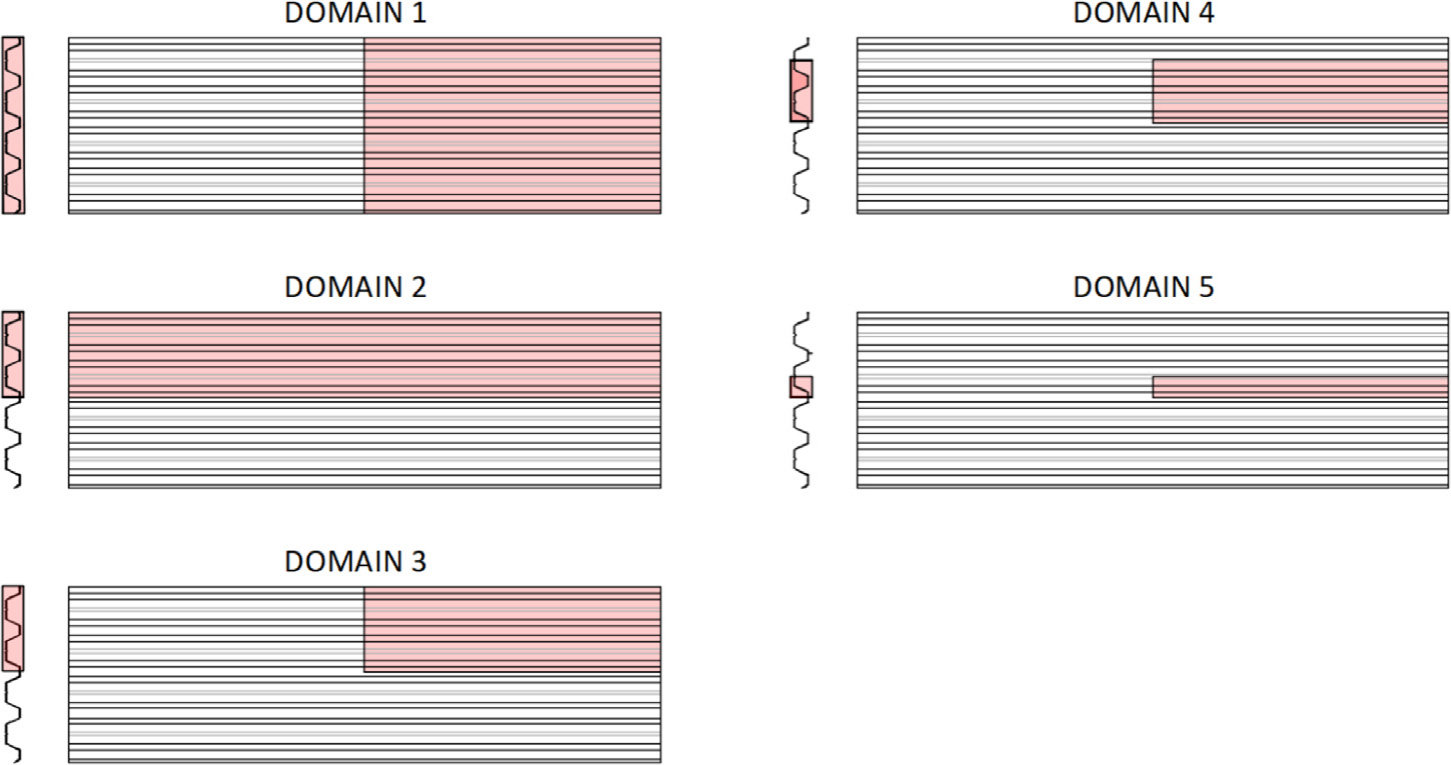
Finite element model domains investigated.

Table 5 shows the sensitivity of the sheet resistance (the SRQ) to the variations in the model domain presented in Fig. 9. Two groups of models are clearly distinguished: (i) those which include the outstanding part of the longitudinal edge rib (cases 1 to 3), and (ii) the models where the outstanding part of the edge rib is cut, or not considered (cases 4 and 5). The experimental tests were conducted following the 2006 edition of Eurocode 3 Part 1-3 [1]. This norm did not explicitly require the removal of the outstanding parts. Consequently, the selected geometrical domain incorporates the full edge. Finally, the one involving less computational cost is chosen: DOMAIN 3. If the tests had been performed according to the current draft of the forthcoming Eurocode 3 Part 1-3 [5], the outstanding parts would have been cut, and the reduced DOMAIN 5 could have been selected.

**Table 5:** Sensitivity to the model domain (*Ratio with respect to DOMAIN 3).

Domain	F_FE_ (N)	Ratio*
1	8145.6	0.98
2 (symmetric imperfection)	8370.0	1.01
2 (antisymmetric imperfection)	8363.2	1.01
**3**	**8293.6**	**1.00**
4	9300.9	1.12
5	9341.7	1.13

#### 4.7.2 Mesh convergence

**Fig. 10:**
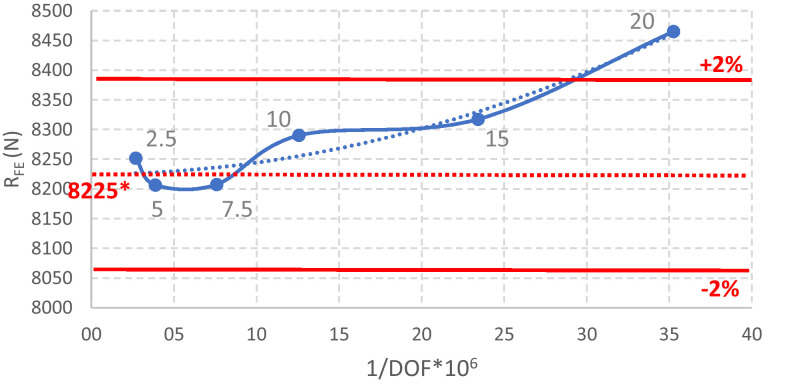
Mesh convergence study using the linear shell element. *Extrapolated value.

**Fig. 11:**
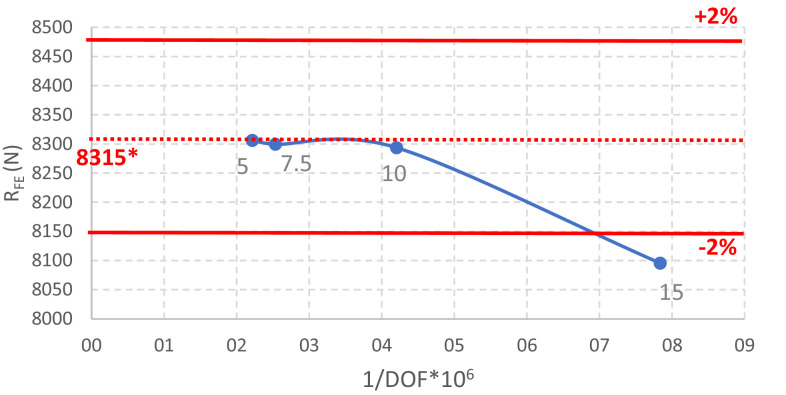
Mesh convergence study using the quadratic shell element. *Extrapolated value.

The mesh convergence study is summarized in Table 6 and Fig. 10, Fig. 11. The finite element mesh finally chosen comprises quadratic elements (SHELL 281), as recommended in [12], with a size of 10 mm and one element in the corners. This mesh yields an accurate error when compared to the extrapolated SRQ: less than 2%, which is in the range accepted by EN1993-1-14 [2]. The experience of the authors is that an error range of 2% is a reasonable limit for simple cold-formed steel members (see also Benchmark Example A.1 in [6]).

**Table 6:** Sensitivity to the finite element type and meshing.

Element type	Element size (mm)	Number of elements in the corners	F_FE_ (N)	Ratio to the extrapolated value
Linear (ANSYS SHELL 181)	20	1	8465.1	1.02
	15	1	8317.4	1.00
	10	1	8290.3	1.00
	10	2	8276.6	1.00
	10	4	8316.7	1.00
	7.5	1	8207.6	0.99
	5	1	8206.7	0.99
	2.5	1	8251.8	0.99
		Extrapolated value	8315.0	1.00
Quadratic (ANSYS SHELL 281)	20	1	8742.9	1.06
	15	1	8095.6	0.98
	10	1	8293.6	1.01
	10	2	8280.8	1.01
	10	4	8282.8	1.01
	7.5	1	8299.4	1.01
	5	1	8306.0	1.01
		Extrapolated value	8225.0	1.00

#### 4.7.3 Imperfection sensitivity

Table 7 displays the sensitivity of the SRQ to the imperfection mode. Concerning the case of single imperfections, modes 1, 2 and 3 of Fig. 8 are considered. Additionally, since the model is very sensitive to the imperfection in the second rib (see first four rows in the Table), two imperfections similar to mode 2 are also included in the study (modes 5 and 6). With regard to combinations of imperfections, several, each addressing different modes in the second rib, have been studied.

**Table 7:** Sensitivity to the imperfection mode and mode combinations (*ratio with respect to combination 123 negative direction).

Mode or mode combination (Fig. 8)	Direction (+ upwards, - downwards)	F_FE_ (N)	Ratio*
1	-	8873.9	1.07
2	-	8357.1	1.01
2	+	8368.0	1.01
3	-	8786.7	1.06
4	-	8864.0	1.07
5	-	8379.1	1.01
5	+	8437.8	1.02
6	-	8415.9	1.01
6	+	8341.9	1.01
1-2-3	**-**	**8293.6**	**1.00**
1-2-3	+	8319.6	1.00
2-3-4	-	8297.7	1.00
2-3-4	+	8334.1	1.00
1-3-5	-	8365.9	1.01
1-3-5	+	8384.5	1.01
1-3-6	-	8356.4	1.01
1-3-6	+	8348.6	1.01
3-4-5	-	8369.2	1.01
3-4-5	+	8396.3	1.01
3-4-6	-	8346.9	1.01
3-4-6	+	8363.0	1.01

It is observed that most of the imperfections and imperfections combinations produce similar results. In the end, the most detrimental one, combination 1-2-3, is chosen for model validation. Additionally, combination 2-3-4 will also be tested for being the second most detrimental combination and for including the distortional mode 4, which is observed in all the numerical failure modes.

### 4.8 Validation

The values shown in Table 3 demonstrate that the proposed numerical model can accurately predict the sheet experimental ultimate force. It is also observed that, as expected from the verification analyses of the previous Section, the two tested geometric imperfection combinations result in very similar final strength predictions. On the other hand, the experimental and numerical force-displacement curves are compared in Fig. 6. (in the legend of the figure, “l” and “r” account for left and right displacement transducers, respectively). The initial stiffness is similar to that measured with the transducers located on the left part of the sheet, which corresponds to the modelled domain (DOMAIN 3 in Fig. 9). However, as the ultimate load is approached, the model becomes slightly more flexible.

Finally, it is pointed out that the experimental failure presented in Fig. 7.a is a bit different from the numerical failure mode displayed in Figs. 7b and c. In the experiments, local buckling is mainly located far from the midspan, while the finite element model indicates that the maximum plastic strains and, consequently, the predicted failure are concentrated in this position. This discrepancy arises from the fact that the model was developed based on imperfection combination 1-2-3, with the highest imperfection amplitudes precisely centered in the sheet, as shown in Fig. 8. If the imperfection combination 3-4-5 is applied, where mode 5 displays maximum imperfection amplitude near the load application zone, the maximum plastic strains shift away from midspan, in a similar way as in the experiments (Fig. 7d). Noticeably, this combination produces almost the same ultimate force as the other previously tested combinations (see Table 3).

## Limitations

The article presents a finite element model example based on tests conducted on a single trapezoidal sheet profile. Readers interested in additional experimental tests on different profiles for further validation of the procedure presented herein are referred to the research project [7] and article [8].

## CRediT authorship contribution statement

**Miquel Casafont:** Conceptualization, Methodology, Software, Validation, Investigation, Writing – original draft. **Frederic Marimon:** Conceptualization, Supervision, Resources. **Oriol Bové:** Investigation, Writing – review & editing. **Miquel Ferrer:** Investigation, Software. **Xavier Centelles:** Supervision, Writing – review & editing.

## Data Availability

Replication Data for: Local buckling of cold formed steel trapezoidal sheets: data for finite element model validation (Original data) (CORA)
